# Health care needs of cancer survivors in general practice: a systematic review

**DOI:** 10.1186/1471-2296-15-94

**Published:** 2014-05-13

**Authors:** Renske A Hoekstra, Marianne J Heins, Joke C Korevaar

**Affiliations:** 1 Netherlands Institute for Health Services Research (NIVEL), P.O Box 1568, 3500 BN Utrecht, The Netherlands

**Keywords:** Cancer survivors, Care needs, Primary health care, General practitioner, Family physician

## Abstract

**Background:**

The number of cancer survivors is increasing due to improved treatments. Consequently, general practitioners will treat more and more cancer survivors in the upcoming years. Only little is known about the care needs of these survivors and guidelines to support general practitioners in their treatment of these patients are lacking. The aim of this study was to gain insight in the health care needs of cancer survivors in general practice.

**Methods:**

A systematic review on cancer survivors’ general practice needs was conducted in PubMed, Embase and the Cochrane Library of Systematic Reviews. Eligible studies could be qualitative or quantitative studies examining cancer survivors’ needs in general practice. Studies of adult survivors, with any cancer type, considered free of active disease and no longer receiving active treatment, were included. For each study a quality score was given using a form developed specifically for this study. Statements about survivors’ general practice needs were collected and corresponding themes were grouped.

**Results:**

Fifteen studies were included, of which twelve were qualitative. Most mentioned general practice needs were psychosocial needs, mainly being support received form the GP, followed by a need for help with medical issues, and a need for information on cancer, recovery, late treatment effects and on adjusting to life after treatment.

**Conclusions:**

Cancer survivors have different types of general practice needs that are currently not or insufficiently met. This review provides a starting point for the development of new guidelines for general practitioners to support in cancer survivorship.

## Background

The incidence of cancer is increasing, which is partly due to the aging population [1]. In addition, because of improved treatment options and detection at an early stage, the five-year survival rate of cancer has increased in recent years [2,3]. Consequently, the number of cancer survivors is increasing. The term ‘cancer survivor’ covers a wide range of patients: from those who have just finished their active treatment period to patients who have been discharged from follow-up for years.

Cancer survivors have more health problems compared to age and sex matched controls [4], which may last for many years after completion of the treatment. These health problems constitute a broad spectrum including infections, chronic diseases, minor illnesses and psychosocial problems, such as sleep disturbance and depression [5]. As a consequence, cancer survivors visit their general practitioner more often than non-cancer controls [6-9]. Despite this higher health care utilization, cancer survivors indicate that they still have health care needs that are unmet [10,11]. Apparently, these patients have specific needs that current aftercare does not meet sufficiently [10,11].

To attain better general practice for cancer survivors, clinical guidelines for cancer survivorship care need to be developed, as reported by the Institute of Medicine and other national councils [12,13]. For this purpose, more clarity on survivors’ health care needs are necessary [14]. Besides care for previously mentioned medical and psychosocial problems, studies in secondary care showed that cancer survivors have needs for informational and emotional support [15-17]. Systematic research on all these needs in general practice is still missing. The purpose of this systematic review is to report how adult cancer survivors describe their care needs in the general practice environment.

## Methods

### Data collection

In September 2012, we searched three databases: MedLine, Embase and the Cochrane Library of Systematic Reviews. The search combined cancer-related terms with terms related to follow-up care, health care needs and general practice (Additional file 1). With ‘care needs’ we mean what cancer survivors want to receive for their health problems, and from whom. Only papers written in English or Dutch were included.

Title and abstract of all articles were independently scanned on inclusion criteria (see below) for eligibility by two researchers (RH, MH). Full texts of all articles rated as potentially relevant were obtained. Authors were approached for articles not available in full text. Full text of the articles was assessed by two researchers (RH, MH) to see whether it met the inclusion criteria. In case the two researchers disagreed about eligibility for inclusion, this was discussed until consensus was reached. If no agreement was reached, a third author (JK) was asked to make a decision.

Inclusion criteria were:

– Original peer-reviewed study (no case reports, review, editorials, letters, conference abstracts etc.)

– Full text obtainable

– Written in English or Dutch language

– Population:

○ consists (mainly) of cancer patients;

○ consists of adults (>18 years);

○ patients are in follow-up care or are not actively treated anymore.

– Results:

○ are obtained by either questionnaires or interviews (individually or in groups);

○ describe (care) needs;

○ contain data about specific needs for general practice.

Papers included

n = 15

Additional studies were identified by reviewing the reference list of all included studies, of reviews found in our search, and by expert referral.

### Quality assessment

As a validated quality assessment instrument for both quantitative and qualitative studies does not exist, as far as we know, we could not use existing checklists to assess the quality of the included studies. We therefore made a checklist based on common elements from existing checklists (Additional file 2) [18-22]. We selected those elements from the existing lists that were applicable to our study.

Two reviewers (RH, MH) independently assessed study quality using the checklist and they resolved differences by discussion. If no agreement was reached, a third reviewer (JK) was asked to make a decision. A quality score was calculated by giving 1 point for each met criterion and dividing this by the maximum obtainable score.

### Analysis and synthesis

We first assembled statements and quotes from the results sections of the qualitative studies concerning survivors’ needs in general practice. We used qualitative synthesis to aggregate and summarise results of the included study. We sought to identify and group overlapping themes and subthemes of needs. The results of the quantitative studies were then categorised into the same themes as those identified in the qualitative studies.

Quality assessment was used to identify the impact of lower scoring studies. We re-analysed our results, using only studies with a quality score higher than 70%, which we considered an acceptable score.

## Results

**Figure 1:**
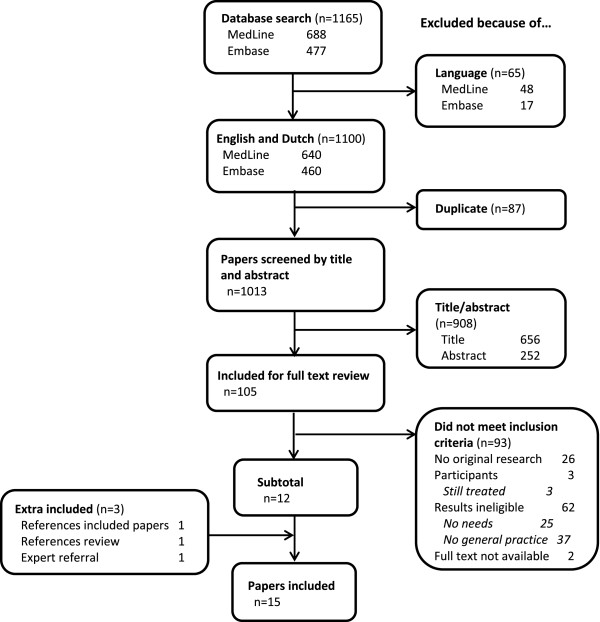
Flowchart of the study selection.

Figure 1 shows the flowchart of the study selection. Finally, 15 studies were included in our study; [23-37] 12 were obtained by database search, the remaining three by expert referral and reviewing references. All included studies were published between 1990 and 2012. Searching in the Cochrane Library revealed two reviews, which were excluded based on the title. Study characteristics are shown in Table 1. Most of the included studies were qualitative studies (n = 12), whereas three studies contained quantitative data from surveys. Of the qualitative studies, most were based on individual semi-structured interviews (66%) with relatively small sample sizes (between 6 and 44 participants). The quantitative studies had considerably larger sample sizes (32–431). Most studies were conducted in a country where many people have a general practitioner and the general practitioner plays an important role as gatekeeper for secondary care, such as the United Kingdom and Denmark. The majority of all participants was between 50 and 80 years old. Although we only included studies with adult participants, we made an exception for Cheung et al [35],. in which the age range started at 16 years. Because the number of participants was relatively high and mean age was 57 years, we decided to include this study.

**Table 1:** Study characteristics

**Author & year**	Study design*	**Nr. of participants**	Mean age at interview**	**Sex: male (%)**	Cancer type**	Specification of cancer type**	**Study country**
**Qualitative studies**							
Eardley 1990 [25]	I	44	71	91	Mix	bladder and prostate	UK
Hudson 2012 [26]	I	42	65 (47–80)	43	Mix	24 breast, 18 prostate	USA
Khan 2011 [30]	I	40	20% <60 25% 61–70 40% 71–80 15% >81	45	Mix	15 breast, 13 CRC, 12 prostate	UK
Rozmovits 2004 [33]	I	39	60 (33–87)	51	CRC		UK
Adams 2011 [24]	I	38	21% <50 37% 50–70 42% >70	50	Mix	9 breast, 6 prostate, 4 CRC, 4 head/neck, 3 lung, 3 melanoma, 2 testis, 2 gynaecologic, 2 Hodgkin, 1 NHL, 1 bladder, 1 renal	UK
Norman 2001 [32]	I	25	58 (28–84)	44	Mix	11 primary cancer sites, most common: breast and lung	Canada
Kantsiper 2009 [28]	G	21	-	0	Breast		USA
Sahay 2000 [34]	I	20	65 (48–87)	-	CRC		Canada
Aabom 2009 [23]	I^^^	16	65 (50–80)	75	Mix	6 rectum, 8 colon, 2 pancreas	Denmark
Kendall 2006 [29]	G	16	53 (35–70)	50	Mix	3 breast, 3 bowel, 3 haematological, 2 prostate, 1 testis, 1 Hodgkin, 1 lung, 2 unknown	UK
Lydon 2009 [31]	G	6	64 (52–73)	0	Ovarian		UK
Jiwa 2006 [27]	G	-	-	-	Breast		
**Quantitative studies**							
Cheung 2009 [35]	-	431	57 (16–91)	27	Mix	216 breast, 43 genitourinary, 39 hematologic, 30 gastrointestinal, 26 head/neck, 21 lung, 17 gynaecologic, 28 other, 21 unreported	USA
Sisler 2004 [37]	-	200	16% ≤49 17% 50–59 30% 60–69 37% ≥70	45	Mix	54 breast, 39 prostate, 24 CRC, 25 lung, 14 reproductive organs, 56 other	Canada
De Padova 2011 [36]	-	32	36 (22–60)	100	Testis		Italy

- = not mentioned/not applicable.

*I = individual; ˆ = together with next of kin; G = group.

**CRC = colorectal cancer, NHL = non-Hodgkin’s lymphoma.

The themes that were identified involved medical, psychosocial and informational needs, need for proactive contact and a group called other. Psychosocial needs were the most frequently mentioned needs; they were mentioned in 12 out of 15 studies (see Table 2). Most studies reported the importance of the general practitioner as a supporter and someone to share ideas and concerns with. The general practitioner was seen as providing “warmth, encouragement and emotional support”, with familiarity as an important aspect of this support [32]. Some studies mentioned that participants were feeling too embarrassed to discuss feelings and problems with a specialist, and rather talked about this with their general practitioner [32,34].

**Table 2:** General practice needs in cancer patients

**Type of need**	Nr. of studies reporting this need*	**Studies**
**Psychosocial needs (total)**	**12**	
Support	7	[23-25,29,31,32,34]
Discussing psychosocial impact of cancer	6	[23,25,29,34,36,37]
Talking about difficulties in relationships	4	[23,28,29,36]
Other subtopics	4	[24,25,27,30]
**Need for medical issues (total)**	**11**	
Non-cancer-related medical problems	7	[25,26,30,32,35-37]
(Late) treatment effects	4	[28,29,34,37]
Other subtopics	5	[24,26,29,35,36]
**Informational needs (total)**	**8**	
Answering questions/general information	6	[24,25,30,31,34,37]
Long-term effects/management	3	[24,30,34]
Peer support groups	3	[24,29,33]
Other subtopics	2	[24,29]
**Need for proactive contact (total)**	**4**	
Proactive contact from general practitioner	3	[24,29,37]
Designated appointment	2	[24,28]
Encouragement to contact the PCP with questions	1	[29]
**Other needs (total)**	**8**	
Financial and practical issues	4	[24,25,28,29]
Referrals to specialists	4	[25,32,34,37]
Care for caregivers and family	4	[27,29,32,37]
Other subtopics	1	[34]

*Subthemes were only listed when they were mentioned in at least one third of the studies that discussed the main theme. For ‘total’, studies that discuss the smaller subtopics not listed in the table, are also included.

Eleven studies outlined the need for help from the general practitioner regarding medical issues. Survivors saw a main role for the general practitioner in treating non-cancer-related medical problems and general preventive healthcare. Regarding cancer-related care they mainly saw a role for the GP in helping with common (late) treatment effects.

The need for information was much-discussed, with a large variety in topics: eight studies reported eleven different information needs. Most described needs were those related to getting information about cancer and recovery: patients spoke about the GP “answering questions” [34,37] and “explaining cancer in lay terms” [24] and “need for more information” [30], in which was not specified which information they would like. Concerning the long-term period, patients wanted information “particularly relating to late effects of cancer treatment” [30]. They also indicated a need to “discuss adjusting to life after treatment” [29] and asked therefore to know more about local peer support groups.

In a small number of studies, four out of 15, cancer survivors mentioned that they would appreciate a proactive approach of their general practitioner, especially shortly after diagnosis or after the end of treatment. This could be either a call or an offer for a contact initiated by the general practitioner.

### Sensitivity analysis

Quality assessment for each study is listed in Additional file 3. Mean quality score was 70%. One study (Eardley et al. [25]) met less than 50% of the quality criteria. Four criteria were unmet in most studies: ‘describing reason for qualitative approach’, ‘describing and considering role of the researchers’, ‘avoiding selection bias’ and ‘describing counterexamples’.

Limiting our analyses to the articles with a score above 70% (8 out of 15) would have influenced our results. Medical and informational needs would be the most mentioned needs, and psychosocial needs would drop down to third place.

## Discussion

### Summary

This study is the first systematic literature review that addresses the care needs of adult cancer survivors in the general practice setting. Cancer survivors’ health care needs in general practice focus mainly on psychosocial support, i.e. discussing the impact of their disease, medical help, mostly for non-cancer related problems, and getting general information about their disease. These are all tasks that a general practitioner is used to perform.

The majority of existing studies on care needs of cancer survivors focused on needs in secondary care or to needs in general. Two literature reviews in secondary care showed a need for psychosocial, medical and informational support [38,39]. Yet, it is not specified in these reviews from whom they want to get this support. Results of these studies are in line with those of our review. This indicates that the type of support that survivors want from their general practitioner covers a large part of their health care needs.

### Strengths and limitations

Our study has some limitations. First, the search strategy was limited by searching in three databases. These are, however, the most prominent in the medical field; it seems therefore unlikely that searching in other databases would have provided many additional articles. Secondly, the search strategy was performed some time ago, so new papers may have been published in the mean time. Third, we might have an under-presentation of all existing needs, since five out of fifteen included articles did not have ‘needs’ as a main topic [23,31,32,34,37]. However, we probably revealed the most important needs by reviewing several articles about this subject.

Another limitation is that the method of quality assessment we used has not been validated, since a validated assessment tool that is both applicable for qualitative and quantitative studies does not exist. Our assessment is, however, based on existing lists, like the checklist from the Critical Appraisal Skills Programme and the RATS guidelines for Biomed Central. The impact of the study quality seems limited, as our sensitivity analyses showed that, although the order of care needs altered after applying stricter criteria, the content of the themes did not change.

We included studies from different countries with a variety of health care systems. These differences may influence expectations and needs regarding general practice, but given the relatively small number of studies on this subject we decided to pool them in our analyses. Post-hoc analyses showed some interesting differences. Studies from the UK, where general practice has an important role in health care, mainly focused on psychological and informational needs. They also frequently mentioned financial needs. Those from Canada, where general practice also has a prominent role, mentioned a large variety of needs. They were the only ones mentioning the need for referral to specialists. In contrast, studies from the USA, where general practice has a less prominent role, mainly mentioned medical needs.

We reviewed qualitative as well as quantitative studies. This posed challenges to the aggregation and description of our results, but it also led to an interesting finding as we found some notable differences and similarities in outcome between these two study types. Need for psychosocial, medical and informational support were mentioned in both study types, but their relative importance differed. In qualitative studies psychological needs were an important topic, while in quantitative studies medical needs were a main topic. These differences are probably due to the study designs. In quantitative studies patients are often provided with a restricted number of options. This enables a more structured needs assessment, but patients may not be able to mention all needs they have. Qualitative studies might provide more options to express specific opinions, which could lead to a larger variety of mentioned needs, and they may also be more suitable to discuss psychological needs.

## Conclusion

We uncovered several domains on which cancer survivors have needs in general practice: medical, psychosocial, informational and proactive contact. Based on the results of this review we cannot say to what extent these needs are currently met, but it could be helpful if general practitioners are aware of these needs of their own patients and thus can adjust to them in the care given during the continuing survivorship period. Stanton *et al.* showed that patients do not spontaneously report all needs and ask all questions to their GP and that few doctors systematically ask for common problems [40]. A more proactive attitude of physicians could help in assessing patients’ needs.

This review also showed that the few studies assessing adult cancer survivors needs in the general practice setting are diverse, both in their quality and study topics. A more systematic way of collecting needs info is needed to guide GPs and their teams in caring for the increasing number of cancer survivors.

## Competing interests

The authors declare that they have no competing interests.

## Authors’ contributions

RH performed the literature search and selection of relevant papers, aggregated results, drafted the manuscript. MH participated in the design of the study, selected relevant papers, aggregated results and helped to draft the manuscript. JK participated in the design of the study, aggregated results and helped to draft the manuscript. All authors read and approved the final manuscript.

## Pre-publication history

The pre-publication history for this paper can be accessed here:

http://www.biomedcentral.com/1471-2296/15/94/prepub

## Supplementary Material

Additional file 1 Search terms. Click here for file

Additional file 2 Quality assessment list. Click here for file

Additional file 3 Quality assessment. Click here for file
