# Supplementary material for: Health care needs of cancer survivors in general practice: a systematic review
Source: BMC Fam Pract. 2014 May 13;15:94. doi: 10.1186/1471-2296-15-94 (PMC4031325; doi:10.1186/1471-2296-15-94)
Supplement: Additional file 1 — Search terms. [file 1471-2296-15-94-S1.doc]

**Additional file 1 – Search terms**

MedLine search terms

#1: cancer OR “neoplasms”[Mesh] OR neoplasm*

#2: survivor* OR “survivors”[Mesh] OR “aftercare”[Mesh] OR aftercare OR “long-term care”[Mesh] OR long-term care OR long term care OR follow up OR follow-up OR long term OR after treatment OR post treatment

#3: needs OR unmet needs OR health care needs OR “health services needs and demand”[Mesh] OR health services needs and demand OR “needs assessment”[Mesh] OR needs assessment

#4: primary care OR family medicine OR “family practice”[Mesh] OR family practice OR “primary health care”[Mesh] OR primary health care OR “Physicians, primary care”[Mesh] OR Primary care physician OR primary care physicians OR “general practitioners”[Mesh] OR general practitioner OR general practitioners OR “Physicians, family”[Mesh] OR Family physician OR family physicians

- #1 AND #2 AND #3 AND #4

Embase search terms

#1: 'neoplasm'/exp OR 'neoplasm' OR neoplasm* OR cancer*

#2: 'aftercare'/exp OR 'aftercare' OR 'after treatment' OR 'post treatment' OR survivor* OR surviving OR 'cancer' NEXT/3 'survivor'

#3: 'needs assessment'/exp OR 'needs assessment' OR needs OR need

#4: 'primary health care'/exp OR 'primary health care' OR 'general practitioner'/exp OR 'general practitioner' OR 'general practice'/exp OR 'general practice' OR 'primary' NEXT/3 'care' OR 'family physician' OR 'family physicians' OR 'primary care physician' OR 'primary care physicians' OR 'family practice'/exp OR 'family practice' OR 'GP' OR 'GPs'

- #1 AND #2 AND #3 AND #4

Cochrane terms (searched in title, abstract, keywords)

#1: neoplasm* OR cancer*

#2: aftercare OR “after treatment” OR “post treatment” OR survivor* OR “long-term care” OR “follow up” #3: needs OR need OR “needs assessment”

#4: “primary health care” OR “primary care” OR “general practice” OR “family practice” OR “general practitioner” OR “general practitioners” OR “family physician” OR “family physicians” OR “primary care physician” OR “primary care physicians”

- #1 AND #2 AND #3 AND #4
