# Supplementary material for: Health care needs of cancer survivors in general practice: a systematic review
Source: BMC Fam Pract. 2014 May 13;15:94. doi: 10.1186/1471-2296-15-94 (PMC4031325; doi:10.1186/1471-2296-15-94)
Supplement: Additional file 2 — Quality assessment list. [file 1471-2296-15-94-S2.doc]

**Additional file 2 – Quality assessment list**

**Article: *(author, year)***

| Scoringsitem | **Answer: yes (Y)/ no (N)/ unclear (U)** |
| --- | --- |
| **Research question**   - Is the research question relevant? - Is the research question clear/ unambiguous? |  |
| **Study design**   - Is described why a qualitative approach is chosen? |  |
| **Participants**   - Is the recruitment of participants clearly described? - Is selection bias avoided as much as possible? - Are the group characteristics described? | -  -  - |
| **Data collection**   - Is a detailed description of interview method given? - Is the location of interviewing described? - Is the duration and/ or the number of interviews described? | -  -  - |
| **Role researchers**   - Is the relationship between researchers and participants described and considered? *(If is stated that plays no role, this question can be skipped)*. | -  - |
| **Ethics**   - Is confidentiality for the participants ensured by either an informed consent or an oral agreement? - Has an ethics committee approved the research plan? | -  - |
| **Analysis**  *Working out*   - Is clearly described how the analysis is done? - Does a theoretical foundation for the analysis exist? - Analysis validated by another researcher? - Was raw data accessible for other researchers?   *Interpretation*   - Did the researchers explicitly describe counterexamples? - Are the interpretations clearly presented and supported by the material from the findings? | -  -  -  -  -  - |
| **Conclusion and discussion**   - Is the conclusion supported by sufficient evidence from the findings? - Are the limitations of the study considered? |  |
| **Others**   - Other comments / remarkable features? |  |
