# Supplementary material for: Health care needs of cancer survivors in general practice: a systematic review
Source: BMC Fam Pract. 2014 May 13;15:94. doi: 10.1186/1471-2296-15-94 (PMC4031325; doi:10.1186/1471-2296-15-94)
Supplement: Additional file 3 — Quality assessment. [file 1471-2296-15-94-S3.doc]

**Additional file 3 – Quality assessment**

Assessment of quality criteria; for all items is asked if it is clearly described.

| **Author & year** | **Research question relevant** | **Reason qualitative approach** | **Recruitment** | **Selection bias avoided** | **Group characteristics** | **Methods of interviewing** | **Location** | **Duration and/or number** | **Role researchers described and influence considered** | **Confidentiality ensured** | **Approval of ethics committee** | **Analysis described** | **Theoretical foundation** | **Analysis validated** | **Raw data accessible for others** | **Counterexamples** | **Interpretation supported by findings** | **Conclusion clear** | **Limitations considered** | **Score (Y)** | **Percentage** |
| --- | --- | --- | --- | --- | --- | --- | --- | --- | --- | --- | --- | --- | --- | --- | --- | --- | --- | --- | --- | --- | --- |
| Aabom 2009 | Y | N | Y | N | Y | Y | N | Y | Y | U | U | Y | Y | U | U | N | Y | Y | Y | 11/19 | 58 |
| Adams 2011 | Y | Y | Y | Y | Y | Y | Y | Y | Y | Y | U | Y | Y | Y | U | N | Y | Y | Y | 16/19 | 84 |
| Eardley 1990 | N | N | Y | N | Y | Y | Y | Y | N | U | U | N | N | U | U | Y | Y | Y | N | 8/19 | 42 |
| Hudson 2012 | Y | N | Y | Y | Y | Y | Y | Y | N | Y | Y | Y | Y | Y | U | N | Y | Y | Y | 15/19 | 79 |
| Jiwa 2006 | Y | Y | Y | N | N | N | N | N | N | U | Y | Y | Y | Y | U | N | Y | Y | Y | 10/19 | 53 |
| Kantsiper 2009 | Y | N | Y | N | Y | Y | Y | Y | Y | U | Y | Y | N | Y | U | N | Y | Y | Y | 13/19 | 68 |
| Kendall 2006 | Y | Y | Y | Y | Y | Y | Y | Y | N | Y | U | Y | N | Y | U | N | Y | Y | Y | 14/19 | 74 |
| Khan 2011 | N | N | Y | N | Y | Y | Y | Y | N | U | Y | Y | Y | Y | U | Y | Y | Y | Y | 13/19 | 68 |
| Lydon 2009 | N | Y | Y | N | Y | Y | Y | Y | Y | Y | Y | Y | Y | Y | U | Y | Y | Y | Y | 16/19 | 84 |
| Norman 2001 | N | Y | Y | N | Y | Y | Y | Y | N | U | Y | Y | Y | Y | U | N | Y | Y | Y | 13/19 | 68 |
| Rozmovits 2004 | Y | N | Y | Y | Y | Y | Y | Y | N | Y | Y | Y | Y | Y | U | Y | Y | Y | N | 15/19 | 79 |
| Sahay 2000 | N | N | Y | N | Y | Y | Y | Y | N | Y | U | Y | N | Y | U | Y | Y | Y | N | 11/19 | 58 |
| **Quantitative studies*** | | |  |  |  |  |  |  |  |  |  |  |  |  |  |  |  |  |  |  |  |
| Cheung 2009 | Y | - | Y | Y | Y | Y | - | Y | - | Y | Y | Y | - | - | U | - | Y | Y | Y | 12/13 | 92 |
| De Padova 2011 | N | - | Y | N | Y | Y | - | Y | - | U | U | Y | - | - | U | - | Y | Y | Y | 8/13 | 62 |
| Sisler 2004 | Y | - | Y | Y | Y | Y | - | Y | - | U | U | Y | - | - | U | - | Y | Y | Y | 10/13 | 77 |

* These are quantitative studies; therefore not all quality criteria are applicable hereto.
